# The neurobiology of moral sense: facts or hypotheses?

**DOI:** 10.1186/1744-859X-12-6

**Published:** 2013-03-06

**Authors:** Donatella Marazziti, Stefano Baroni, Paola Landi, Diana Ceresoli, Liliana Dell’Osso

**Affiliations:** 1 Dipartimento di Medicina Clinica e Sperimentale, University of Pisa, Via Roma 67, Pisa, 56100, Italy

**Keywords:** Morality, Social emotions, Neural networks, Ventromedial prefrontal cortex, Sociopathy, Brain imaging, Frontotemporal dementia

## Abstract

One of the most intriguing frontiers of current neuroscientific research is represented by the investigation of the possible neural substrates of morality. The assumption is that in humans an innate moral sense would exist. If this is true, with no doubt it should be regulated by specific brain mechanisms selected over the course of evolution, as they would promote our species’ survival. In the last decade, an increasing number of studies have been carried out to explore the neural bases of human morality.

The aim of this paper is to present a comprehensive review of the data regarding the neurobiological origin of the moral sense, through a Medline search of English-language articles from 1980 to February 2012.

The available findings would suggest that there might be a main integrative centre for the innate morality, in particular the ventromedial prefrontal cortex, with its multiple connections with the limbic lobe, thalamus and brainstem. The subjective moral sense would be the result of an integration of multiple automatic responses, mainly associated with social emotions and interpretation of others’ behaviours and intentions.

Since converging observations outline how lesions of the proposed neural networks may underlie some personality changes and criminal behaviours, the implications of the studies in this field encompass many areas of the scientific domain.

## Introduction

The issue of the moral nature of man, that had begun in the ancient Greece some thousands of years ago, has been essentially debated within the realm of philosophy, and subsequently of theology and jurisprudence. For a long time morality was considered to be an immaterial concept and, therefore, the possibility of exploring it empirically was postulated only in recent years. The scientific approach to the question stems essentially from the convergence of different lines of research. First, principles seem to exist in our species, those referred to by some authors as “moral or social-moral emotions”, which are linked to the interests and wellbeing of a society or group rather than of single individuals [1]. Second, given that they are fundamental in promoting the group cohesion, it is assumed that they were already present in our primitive ancestors, and that they constituted a crucial factor for the survival of our species (e.g., the importance of food sharing in periods of famine). The feelings of guilt, gratitude and pity are typical examples of emotions with a strong social value.

A great support to the possible neurological bases of moral sense derived from the observations of patients presenting sudden changes in their social interactions as a consequence of cerebral lesions, together with studies on normal and pathological behaviours using neuroimaging techniques [2-4].

Although most of the evidence which will be presented in this paper is still matter of controversy and lively discussion, the possibility is emerging that in the near future a branch of cognitive neuroscience will be devoted exclusively to the study of the biological mechanisms underlying the moral sense [5]. The aim of this paper is to present a comprehensive review of the data regarding the neurobiological origin of the moral sense, together with some theoretical, clinical and legal implications, on the basis of a medline search of English-language articles from 1980 to February 2012 using the following keywords: moral sense, social emotions, neural networks, ventromedial prefrontal cortex, sociopathy, brain imaging, frontotemporal dementia.

### Historical background

Besides the great philosophers, such as Plato, Kant, and Hume, just to mention some of the most distinguished, who raised the question of the nature of the moral sense, one of the first who attempted to study empirically sociopathy, and, therefore, indirectly the moral sense, was Cesare Lombroso, an Italian physician who developed his anthropological theory of delinquency over the course of the five editions of his “L’uomo Delinquente” (“The Delinquent Man”) [6], first published in 1876. After measuring the form and size of the head of several criminals, he concluded that the somatic traits characteristic of these individuals were similar to those of primitive men, and that their antisocial tendencies were present at birth and, therefore, hereditary. Amongst psychiatrists, Philippe Pinel provided one of the most exhaustive descriptions of those forms of behaviours that subsequently were labeled as psychopathic. In 1806 he coined the expression “manie sans delire” (mania without delusion), to underline how this condition was characterized by the presence of cruel behaviours, with no impairment of cognition, perception, or memory [7]. Individuals with this disorder often behave in impulsive and socially unacceptable manners, while being fully conscious of the irrationality and of the destructive consequences of their actions. Independently from Pinel, more or less in the same period, the American physician Benjamin Rush described a similar condition that was labeled as “moral derangement” in his work “Medical Inquiries and Diseases of the Mind” of 1812 [8]. Like Pinel, Rush reported cases of subjects who showed deviant behaviours with no sense of regret, guilt or preoccupations for the negative consequences of their actions, and emphasized the irresponsible and antisocial nature of such individuals. In 1874, Henry Maudsley hypothesized the existence of a specific cerebral centre for moral feelings; according to him, there would be individuals who, since their birth, lack these feelings [9]. In the period between the end of the 19^th^ century to the beginning of the 20^th^ century, in the different editions of his “Psychiatrie: Ein Lehrbuch” (Textbook of Psychiatry) Emil Kraepelin [10] examined extensively the so-called “psychopathic syndrome”. Especially in the second edition of his manual, he underlined how mentally-ill individuals lacking a moral sense may present a congenital deficit to stop or delay their selfish drives. In the fifth edition (1896), for the first time, he defined such a condition as “psychopathic state” and stated that these constitutional disorders could expose such affected individuals to the development of personality disorders over the entire duration of their lives. In the following edition (1899), he considered the psychopathic states to be but one category of the many forms of mental degeneration including also obsessive syndromes and sexual perversion.

Towards the middle of 1800, the case of Phineas Gage, undoubtedly the most famous neurological patient of all times, had great resonance and opened unexpected horizons in the interpretation of the neurological bases of personality [11-13] Phineas Gage was a young man living in New England, working as a foreman in a company that had the task of laying down the tracks for a new railway line. According to his supervisors, Gage was efficient and serious, and could perform his job with extreme precision and concentration: he had, in fact, to carefully organize in advance the position of explosive charges. One day, in 1848, when he was 25 years old, he was involved in a tragic accident when a charge exploded in front of his face. A metal bar entered his left cheek, pushed up through the base of his skull, crossed through the frontal portion of his brain, and went out from the top of his head, falling down at a distance of about thirty meters. Gage was hurled to the ground, dazed and unable to speak, but still conscious. After a few months of convalescence, he had completely recovered physically, and had restored normal hearing and speaking abilities, as well as bodily movements. He only suffered from the loss of vision in his left eye, while his right eye was normal. Unfortunately, as reported by the physicians who took care of him, he had lost “the equilibrium between his intellectual abilities and his animal tendencies”. These changes became overt as soon as the acute phase of the brain lesion had disappeared. Again according to the medical reports of his time, “he was insolent, bizarre, capable of the most vulgar profanity, which he had never used before, showed little regard towards his peers, was intolerant of limitations or advices which were in contrast with his own desires, and was always ready to elaborate plans for future activities that he would shortly thereafter abandon. His language was so obscene that women were advised not to remain too long in his presence”. These new aspects of Gage’s personality were completely at odds with the “moderate habits” and with the “great strength of character” that he had always demonstrated before the accident. His friends and family no longer recognized him. When he returned to his former employers, they refused to hire him: the problem was not in any particular deficiency of ability or in physical capacity, but in his new character. Gage’s story is important because it became evident for the first time that, within the human brain, there were systems that regulated personality and individual behaviours. Brain damage could impair the ethical rules and social conventions, with no impairment of linguistic or reasoning skills. There was something in the brain involved in what are considered peculiarly human characteristics, such as the possibility to anticipate the future and to plan specific actions in a given social context and the sense of responsibility towards oneself and others. The case of Phineas Gage remains emblematic because it highlights how to follow social conventions, behave morally and make advantageous choices require not just knowledge of rules and strategies, but the integrity of specific cerebral systems. Since that time, a series of data have been accumulated to support these preliminary concepts.

**Figure 1:**
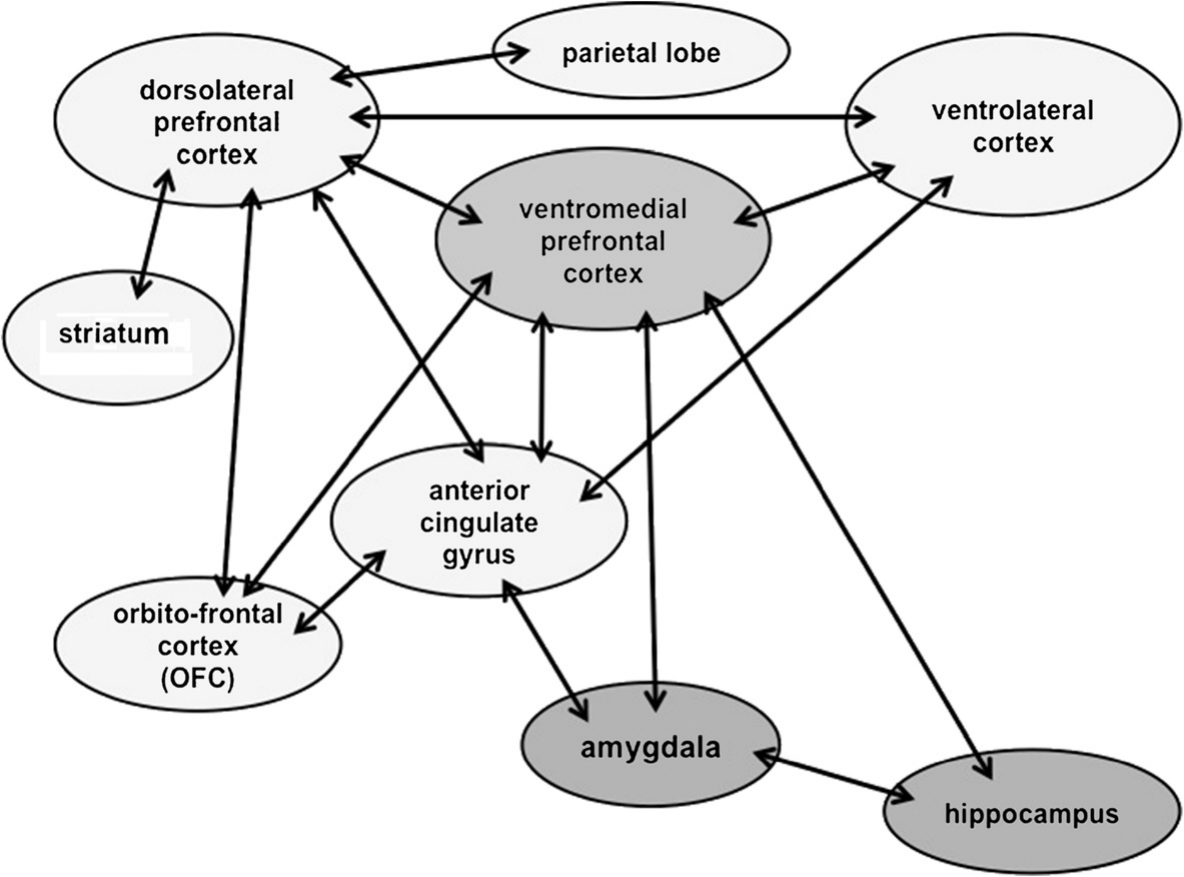
The possible circuits of the “moral” brain, with the ventromedial prefrontal cortex (VMPFC) representing the main integrating centre with all its connections to other cortical, limbic, hypothalamic and brainstem areas.

Besides the evidence coming from the clinical observations in the field of neurology, fundamental data derived also from the studies carried out by Konrad Lorenz in the last century, and from his descriptions of complex social behaviours in animals, which paved the way for the interpretation of several human aspects from an evolutionary perspective [14,15]. It is possible that feelings such as the unwillingness to harm the others, the sense of justice, empathy, and the so-called “theory of mind”, a term defining the capacity to understand the thoughts, feelings, and emotions of the others, developed because of their utility in the survival of man, while promoting cooperation [16-20]. Therefore, it is assumed that an innate morality exists within the human brain, which seems similar to all those mechanisms developed over the course of the evolution that had favored the survival of the individual within a social group. If the moral sense is innate, it should be regulated by specific neural patterns, and therefore it is not surprising that it can be altered by the presence of certain neurological disorders. Much evidence in this sense is derived from functional magnetic nuclear resonance (fMRI) studies in healthy subjects, from neurological data on sociopathic individuals [21-26], and from investigations in patients presenting focal cerebral lesions or frontotemporal dementia (FTD) [27-31]. The contributions of Damasio and coworkers are fundamental in this sense [3,4]. They demonstrated how emotions play a key role in those cognitive processes which imply moral judgment. The process through which moral emotions operate is thought to be mostly unconscious, but the access of these emotions into the consciousness is so rapid that they seem to be strongly rooted in the higher cognitive functions. Therefore, although moral judgment has long been related to verbal and rational processes, the integrity of these processes with a concomitant impairment of the emotional mechanisms, do not warrant moral behaviour in daily life. Interestingly, Damasio and his coworkers set up specific tests for the investigation of patients with minimal brain lesions who showed significant personality and behavioral changes, in the context of normal scoring within traditional neuropsychological investigations [3,4,11]. The emerging data, although still limited, would indicate the presence of an innate brain network for the moral sense, with the right ventromedial prefrontal cortex (VMPFC) representing the main integrating centre with all its connections to limbic, hypothalamic and brainstem areas [32] (Figure 1).

### Definition of moral sense

The moral sense can be defined as that code of values and customs which informs social conduct. In philosophy, it is often divided into “descriptive” and “normative”. This distinction goes back to a debate already present in the pre-socratic era, regarding the possible existence of an universal normative moral sense which would flank descriptive codes proposed by a society, religion, or legal system. Descriptive morality is that code of conduct established by a given society or group which decides what is right and what is wrong: generally, besides not harming the others, it is focussed on the acceptance of authority, and emphasizes the respect of group rules [33]. Normative morality, instead, is that universal code of rules and prohibitions followed by all individuals endowed with reason, above and beyond whatever has been established by the society or the group to which he/she belongs, and is especially centered around the notion of not harming [33,34]. Antigone, in the homonymous Greek tragedy, makes explicit reference to this innate morality when speaking of “laws not written, inalterable… For their life is not of to-day or yesterday, but from all time, and no man knows when they were first put forth.” [35].

Some moral attitudes were likely already present in our hominid ancestors: in particular, it is thought that they were capable of assimilating rules based on a system of reward/punishment, and of attributing and perceiving others’ intentions, feelings, and desires [1,36,37]. The primates closest to man present a range of social behaviours similar to some found in our own species. For example, chimpanzees show an altruistic capacity and a sort of sense of justice, interpreted as antecedents of human morality. There is no doubt that in man the range of emotions and feelings with a moral basis has markedly broadened, and always includes a social component. Just consider faithfulness, shame, embarrassment, gratitude, compassion, pride, the fear of being negatively judged by others, and indignation when coping with unfair behaviours, all emotions which lead individuals to act in a way which is socially acceptable, and which in general have impact upon the surrounding environment [38-40]. These emotions or feelings permit to us to perceive rapidly the moral implications of relationships with others, and, therefore, of acting accordingly to neither diminish nor increase one’s own reputation, in order to obtain greater possibilities of some future social cooperation [41]. According to evolutionary psychology and social neuroscience, moral feelings would be the expression of drives deriving from evolution, such as not harming others, honesty, group spirit, and the sense of authority. Amongst these, the most important might be the aversion to harm and the deep sense of uneasiness when harming another individual [42], along with the sense of justice, shown by the need to punish rebels or those who break the rules [21,43]. On the other hand, moral emotions carried to the extreme and addressed to the preservation of one’s own group, like indignation and contempt, may be at the basis of xenophobia and social conflicts.

### Functional magnetic nuclear resonance (fMNR) studies

The fMNR was utilized only recently to explore the possible neuroanatomical substrates of the moral sense generally in healthy subjects during tasks involving their moral judgment [22-26,42]*.* From these studies it emerged that the brain areas potentially involved would be the (VMPFC, Brodman’s areas, BA 10–12, 25, 32) and the adjacent orbitofrontal (BA 47, portions of BA 10–12 and 25, and BA 44), the ventrolateral cortex (OFC/VL), the amygdala, and the dorsolateral prefrontal cortex (DLPFC) [22-24,26,42]; It is believed that one of the main roles of the VMPFC is to attribute moral and emotional values to social stimuli, anticipate their future outcome, and modulate the mechanism of the theory of mind and empathy, as well as to perceive the others' intentions [11,44-46]. The OFC/VL region would mediate aversive responses related to the social context, modify responses based on feedback, and inhibits automatic-impulsive behaviours triggered by the amygdala [47-49].The amygdala, located in the antero-medial temporal lobes, modulates responses to situations or stimuli perceived as frightening or dangerous, even through the recognition of specific facial expressions [50-53]. The DLPFC would modulate this network, since it is at the basis of the reasoning applied to different moral questions [26]. Furthermore, during some particular tasks in healthy subjects, other brain regions begin to activate in particular the anterior insula [43], the posterior superior temporal sulcus (pSTS) [54-56] the anterior cingulate gyrus [57] the inferior parietal lobes and the temporo-parietal junctions [19,20,54,58] the mesolimbic pathway and the ventral striatum, the precuneus [58] and the posterior cingulate [54-56]. The VMPFC, especially the right one seems to play a fundamental role in the innate moral sense, as it becomes activated during tasks requiring explicit moral judgments, such as the presentation of a “personal” moral dilemma which involves the possibility that the participant may provoke a severe harm to someone [23,59,60] By contrast, the presentation of general moral dilemmas seems to activate mainly the DLPFC [42]. However, a subsequent work would suggest an integration between emotional and cognitive processes in both personal and general dilemmas [26,61].

Besides the ban to harm others, moral feelings serve to reinforce the rules of the group, by attributing a negative judgment to certain actions, and punishing those who do not follow the rules [21,62,63] “Altruistic punishment” is considered a manifestation of the moral desire for justice and fairness, and seems to involve an increased activation of the VMPFC [63,64]. This altruistic punishment is strongly dependent on the fact that others, especially those who carry a bad reputation, deliberately act against the rules [63,65]. Although not directly linked to altruistic punishment, sometimes the “Ultimatum Game”, a neuropsychological test, is used to explore the sense of equity, fairness and justice that are related to altruistic punishment. In this case, a player is asked to divide a sum of money with a second player who can either accept or reject this proposal. If the second player rejects, no player receives anything. If the second player accepts, the money is split according to the proposal [66]. The fMNR scans of individuals playing the Ultimatum Game showed that the VMPFC appears to be involved in the attribution and interpretation of the others’ intentions through their behavior [16]. The OFC/VL (BA47), the anterior insula, and the amygdala, especially the right one [64,67], consequently put into act the altruistic punishment, by eliciting feelings of social aversion and/or exclusion, such as anger, indignation, disgust, and contempt [45,63,68-70]. The theory of mind and empathy are two processes that are strictly related to morality, and these, too, involve the VMPFC, an area which is thought to be involved also in the understanding of the feelings, thoughts and convictions of others [57,71,72]. The cognitive aspect of empathy, such as perceiving another’s point of view and identifying with it (social cognition), involves a part of the VMPFC, especially the areas BA10,11; this is a phylogenetically new system, found only in chimpanzees and in the more highly-evolved mammals [17,18,73-76]. By contrast, the most emotional aspect of empathy, such as the “emotional” contagion, is mediated by the OFC/VL (BA44), through an older neural system [17,74,77-79]. It is hypothesized that certain characteristics, like the perception of the self as an active agent and the evaluation of the similarity between oneself and others, can influence the “cognitive” empathy and the activity of the VMPFC. This suggests that the VMPFC could be involved in the interaction “self/other than self”, or in the influence that the emotional and mental states of one individual may have on those of another [70,80]. When the others' intentions and emotions are internalized or imitated by a subject, other areas activate to modulate the “self/other than self” interaction, probably the mirror neurons of the OFC/VL [74,81-89]. The brain areas engaged by judging others’ emotional states and the forgivability of their behaviours/crimes include left superior frontal gyrus, orbitofrontal gyrus and precuneus. Empathy activates also left anterior middle temporal and left inferior frontal gyri, while forgiveness activates also posterior cingulate gyrus and right caudate nucleus [90-92]. In addition, the right temporal-parietal junction, previously implicated in reasoning about others’ thoughts, beliefs and intention in moral and non- moral contexts, seems to be activated in mitigating blame for accidental harms and, therefore, in forgiveness [93].

In the case that the “self” is threatened by the superiority of others (envy) [57], there is the involvement of the anterior cingulate area, or of the ventral striatum when the pleasure is derived from the misfortune of others (only in the German language there is a term, “Schadenfreude”, to indicate this feeling). As far as mirror neurons are concerned, this is a class of neurons which selectively are activated by both when an action is carried out by an individual or he/she observes that action being performed by others. The neurons of the observer “mirror” what is taking place in the mind of the observed subject, as if it were the observer that was carrying out the action [76,94]. The areas activated during the observation of behaviour of the other individuals are the anterior rostral portion of the inferior parietal lobe, the inferior part of the anterior central gyrus, and the posterior part of the inferior frontal gyrus. In some cases, the activation of the anterior area of the inferior frontal gyrus and of the dorsal pre-motor cortex have been reported. The ability of the human brain to self-activate when the emotions of others are perceived, expressed through facial mimicry, gestures, and the tone of voice, and the ability of immediately decoding this perception in “visceromotor” terms, enables every individual to act according to the so-called “empathic participation” [59,83]. This represents a form of bio-social behaviour, prior to linguistic communication, that characterizes and triggers inter-individual relations, which are at the basis, perhaps, of all social behaviours. It must be underlined, however, that, as fascinating as all this might be, these are only hypotheses, given that mirror neurons have been found only in motor areas.

### Brain areas and sociopathic behaviour

Generally sociopathic individuals are defined as those lacking a sense of morality, empathy, regret or guilt for their actions, who feel little or no pity, or who manifest a cold and calculated aggression, without alterations of their higher cognitive processes [69,95]. Generally, they show limited alterations in cardiac frequency, in skin conductance or in respiratory activity, when they look at frightening or unpleasant pictures, and they demonstrate other blunted responses of the autonomic nervous system, when facing the others' suffering, as well as an impairment to recognize sad or frightened expressions [96-99]. Studies aimed at exploring possible brain abnormalities in criminals are quite limited, and, in any case, must be considered with cautions, given the small size of the samples, their heterogeneity, and the presence of potential-confounding factors, such as drug abuse. A high percentage (two-thirds) of a sample of murderers fulfilled the criteria for a neurological diagnosis, such as brain trauma, mental retardation, cerebral paralysis, epilepsy, and dementia [100]. Further, some criminals often display unspecific alterations at temporal level, or disturbance of other brain areas which can be detected by electroencephalogram or through more sophisticated neuroimaging techniques [101]. In institutionalized patients, the use of neuropsychological tests permitted to observe deficits of some frontal functions, such as an inability to modify one’s own responses (response reversal learning), or to inhibit risky behaviour following a negative feedback [102-107]. Moreover, in a group of murderers who had been convicted not guilty for mental infirmity, or in violent psychiatric patients, hypometabolism and hypoperfusion of the frontotemporal areas have been described [97,101,103,108-110]. Voxel-based morphometry, a technique based on a systematic comparison of the values of pixels amongst different subjects, seems to suggest a correlation between the reduction of gray matter at both frontopolar and OFC/VL level and an increase in the degree of psychopathy [111,112]. Sometimes, a decreased volume of prefrontal gray matter associated with a lower level of autonomic activation has been described in criminals responsible for cruent acts [95]. Further, the more reduced is the volume of the prefrontal cortex, the greater is the tendency towards antisocial behavior [113]. Non-affective and insensitive children seem to present a greater amount of gray matter in the medial frontal regions, which seems to suggest a delay in cortical maturation [114]. Besides the abnormalities in the frontal lobe, some sociopathic individuals show a reduced functionality of the amygdala [115] which, as already noted, modulates anxiety and fear responses. Such an information is necessary for the development of socialization based on moral principles [69,116,117], as well as the recognition of the emotional value of sensorial experiences and face expressions [50]. In fact, animal studies show that an early amygdala dysfunction may block the normal development of the VMPFC and of the OFC/VL. It is, therefore, hypothesized that in some forms of sociopathy, early amygdala alterations could provoke dysfunctions of the VMPFC and of the OFC/VL, which would lead to an erroneous association between actions that are harmful to others and negative reinforcement of the discomfort of the victim.

### Studies on patients with brain lesions

The story of Phineas Gage, described above, is currently a milestone in neurology, as it demonstrated for the first time, and unequivocally, that moral judgment requires the integrity of specific cerebral systems. Thanks to recently developed techniques, it was possible to better explore the cerebral areas mainly involved in the Gage's case, in particular the VMPFC [11]. Nowadays the contributions coming from studies on patients whose clinical conditions are similar to those of Gage showed that deficit involving VMPFC and the nearby OFC/VL [98,118] may modify the moral sense. Furthermore, some evidence indicates that alterations of the right frontal lobe can be associated with some abnormal social behaviours, while those of left frontal lobe can be linked to outbursts of anger and violence [119,120]. Focal lesions of the VMPFC and of the OFC/VL interfere with the normal development of moral sense and judgment and, moreover, if they occur before 16 years of age, they can lead to severe antisocial behaviour, insensitivity to the future consequences of decisions, and to the repeated failure of attempts to correct aberrant behaviours [121,122]. Patients with focal lesions of the VMPFC, especially of the right one, show indifference in front of violations of socio-moral rules, and little empathy towards the victims [45,73,109,123-130]. Lesions of the VMPFC may impair feelings of pity, shame, guilt, envy, unjustified pride, and malice, all involved in one’s own “point of view” and that of others [121,131,132]. Although the concept of the theory of mind remains intact, such individuals cannot understand the others' feelings and emotions (theory of the affective mind), as it emerges from tests regarding the sense of irony and the gaffes [126,133]. Patients with lesions of the VMPFC show low or no autonomic responses (such as heart rate, skin conductance, pupillary reactivity, piloerection, sweating, etc.), especially to social stimuli [129,134]. In addition, they appear to be fake, manipulative, and aggressive. Finally, lesions of the OFC/VL alter both the use of immediate feedback coming from social signals and emotions, and the control of emotional and impulsive responses [47,68,126,128,132,135-138]. Subsequently, more specific tests designed to explore the formulation of moral judgments in subjects with lesions in the VMPFC were utilized. A comparison of seven patients with 12 control subjects regarding personal, impersonal, and non-moral dilemmas demonstrated that the former had a greater propensity to judge violations of personal morals as acceptable behaviour, and they did so with extreme rapidity and certainty [124]. In a similar study, six patients with bilateral focal damage of the VMPFC were examined [126]: they presented a low level of autonomic activation in response to emotionally-charged images, and displayed limited empathy, sense of embarrassment and sense of guilt. In both these studies, the subjects tended to make utilitarian choices when facing with moral dilemmas. These results were recently confirmed in patients with lesions of the VMPFC, whose variations in skin conductance were also evaluated as indicators of the emotional state. These patients, in contrast to the control subjects, chose solutions with a personal advantage and detriment of others, with no variation of skin conductance during the formulation of their moral judgment. It was, thus, hypothesized that the VMPFC is widely involved in the modulation of moral judgment and in anticipating the emotional consequences of the rule violation [124,126]. It was further reported that these patients continue to refuse unfair offers during the “Ultimatum Game” test [139]. This suggests that when the OFC/VL region is integral, it may be at the basis of feelings of social aversion, and can continue to apply altruistic punishment in situations in which fairness and intentionality are clear or predefined. Although recent lesions at the level of the VMPFC can alter the acquisition of a moral sense [129,130], subsequently the patients maintain an intact moral reasoning, and conserve their awareness of rules and moral conventions [139-142]. These individuals show a deficit of pro-social feelings, and cannot use their ability moral reasoning to anticipate the consequences and feelings associated with their actions [123,143-147]. It is noteworthy to mention that split-brain patients judge moral violations on the basis of the outcome. This is generally explained by the possibility that the left hemisphere, that responds verbally to the dilemmas, does not receive inputs from the right temporoparietal junction, possibly implicated in belief attribution [58].

### The model of frontotemporal dementia (FTD)

Although a number of brain disorders, such as Huntington’s disease, traumas, and some frontal tumors, can modify socio-moral behaviours, with no doubt the most important of these is FTD, given that approximately 50% of the affected patients present sociopathic behaviours. FTD belongs to a family of non-Alzheimer’s degenerative dementias, mainly associated with atrophy of the frontal lobes and of the anterior portion of the temporal lobes. They are characterized by behavioral, personality, social conduct, and verbal expression disorders, with a relative maintenance of memory and of topographical orientation. In contrast to the cognitive and memory deficits typical of Alzheimer’s dementia, the main symptoms of FTD consist in the violation of the previously-acquired social norms, and also of sociopathic behaviours, loss of empathy, and loss of the perception of feelings and of the awareness of one’s own behaviour and of its consequences [148]. The involution of the right frontal area is associated with unpleasant social behaviour and altered perception of the feelings and intentions of others [27-30,149]. These patients begin to violate social and moral rules during the early stages of the disease, and commonly a lot of them demonstrate a reduction of their sense of tact and decency (including improper physical contacts), and of their verbal and non-verbal communication [150]. Inappropriate or transgressive sexual contacts, violence, and aggression are similarly frequent [151-155]. These sociopathic forms of behaviour are often associated with alterations of the right frontal lobe, perhaps at the level of the VMPFC, as revealed by imaging studies [28,150]. Furthermore, some FTD patients show a greater deficit in the immediate response to moral dilemmas, compared with patients affected by Alzheimer’s or to control subjects [156]. By utilizing the relatively intact processes of DLPFC, FTD patients resolve moral dilemmas in a cold, logical, and calculating way. Investigations of their personality highlighted a decreased empathy when the right OFC/VL is involved, and interpersonal coldness or reduced emotional empathy in case of severe alterations of the anterior temporal lobe [157-159]. Moreover, FTD patients show a particular deficiency in their ability to calibrate the entity of moral violation, as well as social concepts themselves, especially when the right anterior temporal lobe is involved [136,146,160,161]. The lack of moral emotions and of the sense of union between themselves and the others could explain their impaired moral judgment, as well as their antisocial behaviours. A selective impairment of making decisions relative to personal moral judgments is often seen, notwithstanding a relatively preserved capacity for moral reasoning [156]. Impersonal responses to violations of personal morals lead to the hypothesis of an early neuropathological focus localized in the VMPFC [150]. These characteristics, associated with insufficient control over impulsivity, due to the involvement of the nearby OFC/VL, can provide an explanation of the tendency of these patients towards sudden violations of morals, while maintaining a full awareness of the consequences. Because of the early onset of psychic and behavioral disorders, especially at the beginning, FTD can be confused with a psychiatric disorder. The neuropsychological approach, with its characterization of different cognitive domains (memory, language, executive function, praxic and visuospatial ability), is fundamental for the diagnosis. The second diagnostic level is represented by neuroradiologic evaluation, with the detection of a symmetrical or asymmetrical atrophy in the frontal and temporal lobes, while functional neuroimaging is fundamental to diagnose cases with initial cerebral atrophy [53,162].

## Discussion

Different lines of research suggest that humans are equipped by an innate morality, the so-called called normative moral sense, that would result from a neural network including different brain regions, with the main centre represented by the VMPFC, especially the right one. A moral judgment and behaviour require, in fact, the integration of different processes: the decoding of signals perceived by the sensitive organs (thalamus), the activation of basic emotions (anteromedial temporal lobe, brain stem, and the nuclei of visceromotor centres), the awareness of the relevance and importance of the stimuli (VMPFC and OFC), and the implementation and control of potentially related forms of behaviours (frontal lobes) [23]. According to this model, an alteration of one of the cortical or subcortical centres could underlie changes in social behaviour.

The VMPFC, closely connected to the limbic system, mediates automatic reactions which are evident when one has to face moral violations [23,26,82]. Brain lesions or disorders involving the right VMPFC seem to mitigate moral emotions and responses to dilemmas concerning both harming the others and the sense of fairness and justice. In fact, patients with such lesions may show different alterations in their emotional functioning, which include affective apathy, reduction of empathy, emotional weakness, and difficulty in controlling anger and frustration. The OFC/VL can control the aversive emotions inherent to the social sphere, inhibit immediate responses coming from the amygdala, and suppress impulsive behaviour and disinhibition [47,163]. The amygdala mediates response to fear/anxiety, disgust, and negative social stimuli, and modulates the understanding of moral or social boundaries, as well as of certain facial expressions, mainly negative [50]. The lack of reactivity to some stressful stimuli recorded in some psychopathic subjects has been attributed to amygdala lesions. More extensive bilateral lesions involving also the adjacent anterior temporal cortex underlie the Klüver-Bucy syndrome, characterized by an exaggerated tendency towards the oral and tactile exploration of objects, hypersexuality, bulimia, absence of fear, increased aggression, memory deficit, and difficulty in recognizing people and objects [164,165].

A few studies have proposed that this moral network could be bypassed by rational processes mediated by the DLPFC, leading to utilitarianism, that is to say, acting for the greater good of the greatest number of people [45]. The result is the creation of a positive psychological tension (empathy?) which permits to understand the mental state of the other (theory of mind) [60]. Unless it is actively inhibited, the initiation of this process occurs automatically through the mirror neurons, and results in empathy, emotions, and moral behavior [166-169]. These new findings, although still preliminary, have a number of implications. For example, when an individual presents an unusual behaviour for the first time, or a complete personality change compared to a previous model of behaviour, the possibility of an underlying neurological disorder should be taken into account [170]. Similarly, family members and acquaintances should also be informed of the possibility that abnormal behaviour can sometimes be independent from the willful control of the patient. Furthermore, pharmacological treatments exist for controlling impulsivity. A discussion of the pharmacological management of these impulsive behaviours falls beyond the scope of this review: it should be only mentioned that a certain effectiveness has been demonstrated for selective inhibitors of serotonin reuptake (SSRIs), beta-blockers, and mood stabilizers, such as valproate, carbamazepine, lamotrigine, and topiramate.

## Conclusions

In the past few years, the concept has emerged that there may exist an innate moral sense which would be at the basis of those emotions, feelings and behaviours typically human aiming at promoting group cohesion and cooperation. A specific neural network for this innate moral sense has been proposed on the basis of clinical observations showing how alterations in this network could, in part, explain certain forms of deviant, sociopathic, or criminal behaviour. Such findings have been also supported by brain imaging studies in healthy subjects.

Although preliminary, the available data would seem to suggest that the problem of some forms of criminality may be rooted in brain alterations. On the other hand, we cannot invoke only these latter to justify a vicious act, or to eliminate that personal responsibility which must be always taken into account.

In any case, this is an emerging area that only recently has become a topic of neuroscientific investigation and several questions are still unanswered and need to be addressed.

If traditional theories underlined the role of higher cognitive processes, latest work stressed the role of emotions [1], with moral sense deriving from the perfect integration and integrity of rationality and emotions [4][26][61]. Therefore, according to these last authors, both cognitive and emotional processes “play a crucial and sometimes mutually competitive roles” in the emergence of moral judgment. Indeed, utilitarian judgments, mainly regulated by cognition, requires emotions to be motivated, and the opposite is true for non-utilitarian judgements [171]. However, there is an urgent need of brain imaging data contributions based on specific tasks exploring and assessing the different components of morality.

There also exists another open and great question in this area regarding the role of environmental influences and, in particular, of the primary experiences of attachment, education and of interpersonal relationships in modulating the organization of the moral sense.

The hope is that future neuroscientific research will provide support to these notions that remain still theoretical, although the horizons that they open up are fascinating and not limited to the scientific domain.

## Competing interests

The authors declare that they have no competing interests.
